# Systemic Maternal Inflammation and Neonatal Hyperoxia Induces Remodeling and Left Ventricular Dysfunction in Mice

**DOI:** 10.1371/journal.pone.0024544

**Published:** 2011-09-14

**Authors:** Markus Velten, Kirk R. Hutchinson, Matthew W. Gorr, Loren E. Wold, Pamela A. Lucchesi, Lynette K. Rogers

**Affiliations:** 1 Center for Perinatal Research, The Research Institute at Nationwide Children's Hospital, Department of Pediatrics, The Ohio State University, Columbus, Ohio, United States of America; 2 Center for Cardiovascular and Pulmonary Research, The Research Institute at Nationwide Children's Hospital, Department of Pediatrics, The Ohio State University, Columbus, Ohio, United States of America; University of Dayton, United States of America

## Abstract

**Aims:**

The impact of the neonatal environment on the development of adult cardiovascular disease is poorly understood. Systemic maternal inflammation is linked to growth retardation, preterm birth, and maturation deficits in the developing fetus. Often preterm or small-for-gestational age infants require medical interventions such as oxygen therapy. The long-term pathological consequences of medical interventions on an immature physiology remain unknown. In the present study, we hypothesized that systemic maternal inflammation and neonatal hyperoxia exposure compromise cardiac structure, resulting in LV dysfunction during adulthood.

**Methods and Results:**

Pregnant C3H/HeN mice were injected on embryonic day 16 (E16) with LPS (80 µg/kg; i.p.) or saline. Offspring were placed in room air (RA) or 85% O_2_ for 14 days and subsequently maintained in RA. Cardiac echocardiography, cardiomyocyte contractility, and molecular analyses were performed. Echocardiography revealed persistent lower left ventricular fractional shortening with greater left ventricular end systolic diameter at 8 weeks in LPS/O_2_ than in saline/RA mice. Isolated cardiomyocytes from LPS/O_2_ mice had slower rates of contraction and relaxation, and a slower return to baseline length than cardiomyocytes isolated from saline/RA controls. α-/β-MHC ratio was increased and Connexin-43 levels decreased in LPS/O_2_ mice at 8 weeks. Nox4 was reduced between day 3 and 14 and capillary density was lower at 8 weeks of life in LPS/O_2_ mice.

**Conclusion:**

These results demonstrate that systemic maternal inflammation combined with neonatal hyperoxia exposure induces alterations in cardiac structure and function leading to cardiac failure in adulthood and supports the importance of the intrauterine and neonatal milieu on adult health.

## Introduction

The impact of maternal health and the neonatal environment on the development of adult cardiovascular disease has recently been appreciated. Most notable are the investigations by Barker and coworkers [1] correlating low birth weight and increased cardiovascular mortality in adulthood. Since the first observations by Barker [2], several studies have expanded this association with low birth weight to include the development of hypertension, insulin resistance, and coronary artery disease. Furthermore, epidemiological studies suggest that factors leading to adult cardiovascular diseases are already present during childhood.[3], [4]

A substantial portion of cardiovascular disease cannot be directly correlated with common risk factors or preexisting diseases, implicating a more subtle origin as the pathologic source. Studies focusing strictly on low birth weight have not revealed precise risk factors in humans. Furthermore, animal models have not identified specific mechanisms for the influences of birth weight on adult health.[5], [6], [7] Consequently, the impact of intrauterine and early neonatal influences on developmental programming due to low birth weight or early gestational age and cardiovascular health warrants further investigations.[7]

Preterm birth and thus low birth weight occurs in approximately 12% of the population and results from a broad range of diverse conditions often brought on by poor maternal health or inflammation.[8] Systemic maternal infections or sources of inflammation such as periodontal [9], urinary tract [10], or respiratory infections [11] are often ignored in the context of fetal development. However, the fetus is exposed to increased expression of cytokines, chemokines, and/or lipid mediators through the circulation as a result of maternal inflammation.[12], [13]

*In utero* exposure to maternal inflammatory mediators is likely to impact the fetus and can result in fetal programming, either physiologically or epigenetically. In addition, chronic inflammatory conditions can also negatively impact the fetus and adversely affect neonatal outcomes, specifically increasing the incidence of preterm birth.[8], [14], [15] Recently, animal models have demonstrated that maternal hypercholesterolemia can alter arterial gene expression, vascular reactivity, and cause endothelial dysfunction, accelerating atherosclerosis in the offspring.[6], [16] Other animal models have demonstrated an association between maternal undernutrition, hypoxia exposure, and the development of hypertension.[14], [17], [18]

The intrauterine effects are often compounded by the events associated with birth and the implementation of life sustaining medical interventions such as oxygen administration during the perinatal period. Animal studies have demonstrated effects of maternal hypoxia on cardiovascular development in the offspring. Specifically relevant are the findings that hypoxia interferes with inotropic stimulation and changes the sensitivity of adrenergic receptors resulting in permanently altered responses [19], [20], [21], [22]. Conversely, hyperoxia exposure also poses a threat to the developing cardiovascular system and negatively impacts the neonate. Yzydorczyk et al. have identified changes in systolic and diastolic blood pressure and increased resting heart rates in adult rats following neonatal exposure to hyperoxia.[23] Most recently, Seehase et al. reported that antenatal exposure of fetal sheep to endotoxinaemia resulted in cardiac inflammation and dysfunction within 3 days.[24] However, the consequences of neonatal hyperoxia exposure on developing organ systems other than the lung have not been extensively investigated. The combined effects of maternal inflammation, preterm or small infants, and postnatal interventions such as hyperoxia are likely to have profound effects on offspring, making them vulnerable to the development of adult diseases.

In the present study, we hypothesized that the combination of systemic maternal LPS administration and medical interventions such as neonatal hyperoxia exposure would alter cardiac development, impairing function later in life. Our model of systemic maternal inflammation and neonatal hyperoxia exposure offers a novel approach to investigate the influence of the neonatal environment on the developing cardiovascular system and may provide new insights into the etiology of adult heart failure.

## Results

### Body weights and LV weights at 8 weeks of age

Maternal inflammatory response to LPS was assessed in lung tissue from pregnant dams 4 h after i.p. LPS or saline injection on embryonic day 16 (E16). LPS injection induced a robust TNFα (saline 1.10±0.31 vs. LPS 6.57±0.68) and IL1-β (saline 1.26±0.61 vs. LPS 6.90±1.47) mRNA increase in the lungs of pregnant dams. To investigate in the duration of the maternal inflammatory response to a single LPS injection we assessed KC protein levels in maternal serum on E17 and E19. LPS injection significantly increased KC levels in maternal serum on E17 (saline 0.39±0.15 ng/ml vs. LPS 1.51±0.28 ng/ml) and E19 (saline 0.38±0.23 ng/ml vs. LPS 0.89±0.22 ng/ml). All saline injected and 80% of the LPS injected dams gave birth at term. No differences were observed in litter sizes (saline 6.7±0.2 vs. LPS 6.3±0.4 pups) or gender distribution between saline or LPS injected dams that gave birth.

Body weights were not different through the first 2 weeks of life (data not shown). However, at 8 weeks of age, body weights were significantly lower in LPS/O_2_ compared to saline/RA exposed mice, while each single exposure (saline/O_2_ or LPS/RA) had no effect (Table 1). Histologically there were no gross differences in heart structure or size of the LV or RV. LV wet weights were lower at 8 weeks of age, in LPS/O_2_ than in saline/RA mice, while single exposures had no effect (Table 1). After normalizing LV wet weight to body weight, LPS/O_2_ mice still exhibited a lower LV to body weight ratio compared to the saline/RA mice. However, absolute liver weights or liver weights normalized to body weights were not different between groups (Table 1).

**Table 1:** Body, left ventricle, and liver weights from 8 week old mice after prenatal saline or LPS and neonatal RA or hyperoxia exposure.

	Saline RA	Saline/O_2_	LPS/RA	LPS/O_2_
body weight (BW) [g]	24.29±0.50	22.48±0.55	23.04±0.50	20.76±0.49 *
left ventricle (LV) [mg]	75.59±1.60	69.89±2.76	74.67±2.07	63.89±1.65 *
LV/BW	3.14±0.08	2.99±0.09	3.10±0.06	2.86±0.08 *
liver weight [g]	1.413±0.05	1.319±0.05	1.304±0.04	1.260±0.04
liver/BW	60.02±2.47	60.97±2.37	58.06±1.28	60.98±1.53

Data were analyzed by one-way ANOVA followed by Bonferroni post hoc. n = 21–24 mice per group p<0.05 compared to saline/RA exposed mice.

### Echocardiography

**Figure 1:**
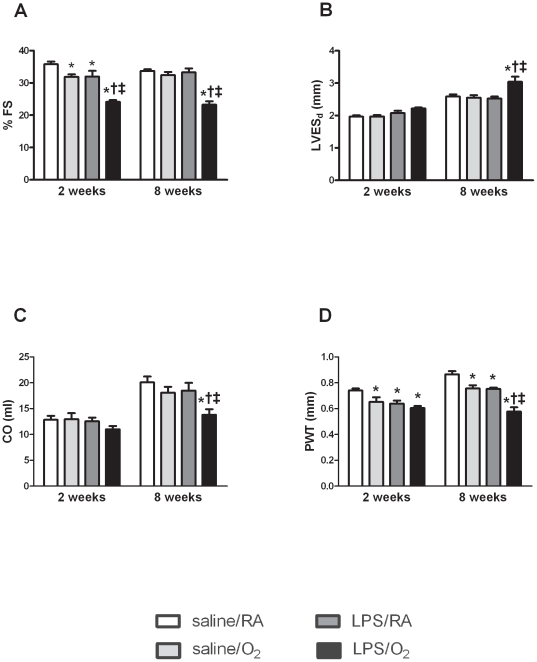
Functional and morphological parameters achieved by M-mode echocardiography of mice exposed to maternal saline or LPS on E16 and 14 days of neonatal RA or O_2_ at 2 and 8 weeks of age. Data were analyzed using two-way ANOVA and Bonferroni post hoc, n = 8–9 mice per group, p<0.05 compared to saline/RA (*), saline/O_2_ (†), or LPS/RA (‡) exposed mice.

Echocardiographic parameters of LV structure and function were assessed at 2 and 8 weeks of age (Figure 1 and Table 2). At 2 weeks of age, fractional shortening (FS) was modestly lower in the saline/O_2_ and LPS/RA than in the saline/RA exposed mice but severely decreased in LPS/O_2_ exposed animals (Figure 1A). Furthermore, FS remained lower in LPS/O_2_ exposed mice at 8 weeks of age, while LV systolic function was not different in saline/O_2_ and LPS/RA exposed mice compared to saline/RA controls (Figure 1A). No difference was found in LV end diastolic diameter (LVED_d_) in any of the groups (Table 2). However LV end systolic diameter (LVES_d_) tended to be greater at 2 weeks and was increased significantly at 8 weeks of age in LPS/O_2_ expose mice compared to all other groups (Figure 1B). Cardiac output (CO) was slightly but not significantly lower at 2 weeks but was significantly decreased at 8 weeks of age in LPS/O_2_ compared to single treated or saline/RA exposed mice (Figure 1C). All treatments significantly decreased posterior wall thickness (PWT) at both time points, although PWT was even lower in LPS/O_2_ compared to saline/O_2_ and LPS/RA exposed mice at 8 weeks of age (Figure 1D). Ejection fraction (EF) was lower in LPS/O_2_ exposed mice at 2 and 8 weeks of age compared to all other groups. At 2 weeks of age LV end systolic volume (LVESV) was greater in LPS/O_2_ exposed mice compared to both saline treated groups and at 8 weeks of age greater than all groups. Stroke volume (SV) was lower at 8 weeks of age in LPS/O_2_ exposed mice compared to controls. No differences were observed in heart rate (HR) or LV end diastolic volume (LVEDV) at any time point (Table 2).

**Table 2:** LV end diastolic diameter (LVEDd), Ejection Fraction (EF), LV end systolic volume (LVESV), LV end diastolic volume (LVEDV), stroke volume (SV), and heart rate (HR) at 2 and 8 weeks of age.

2 weeks	saline/RA	Saline/O_2_	LPS/RA	LPS/O_2_
LVED_d_ [mm]	3.06±0.06	2.89±0.05	3.08±0.06	2.93±0.04
EF [%]	66.76±1.11	61.36±1.23	61.26±2.59	50.36±1.21 *†‡
LVESV [µl]	12.32±0.67	12.44±0.62	14.20±1.40	16.45±0.61 *†
LVEDV [µl]	37.05±1.81	32.14±1.14 *	36.02±0.63	33.09±1.00
SV [µl]	27.18±1.45	26.40±2.16	27.17±1.49	22.38±1.53
HR [bpm]	471.3±5.72	487.5±8.17	465.0±5.65	494.0±8.00

8 weeks	saline/RA	Saline/O_2_	LPS/RA	LPS/O_2_
LVED_d_ [mm]	3.90±0.08	3.77±0.07	3.91±0.20	3.9±0.2
EF [%]	63.07±0.73	61.20±1.38	62.42±1.65	44.54±1.61 *†‡
LVESV [µl]	24.86±1.46	23.80±1.70	23.11±1.37	36.23±4.75 *†‡
LVEDV [µl]	66.46±3.07	61.29±2.95	62.19±3.71	67.06±7.01
SV [µl]	43.10±0.82	40.11±2.36	41.53±3.71	31.58±2.71 *
HR [bpm]	468.2±14.9	448.0±10.3	450.0±6.6	439.5±8.3

n = 8 (4 males, 4 females), p<0.05 compared to saline/RA (*), saline/O_2_ (†), and LPS/RA (‡).

### Cardiomyocyte function

**Figure 2:**
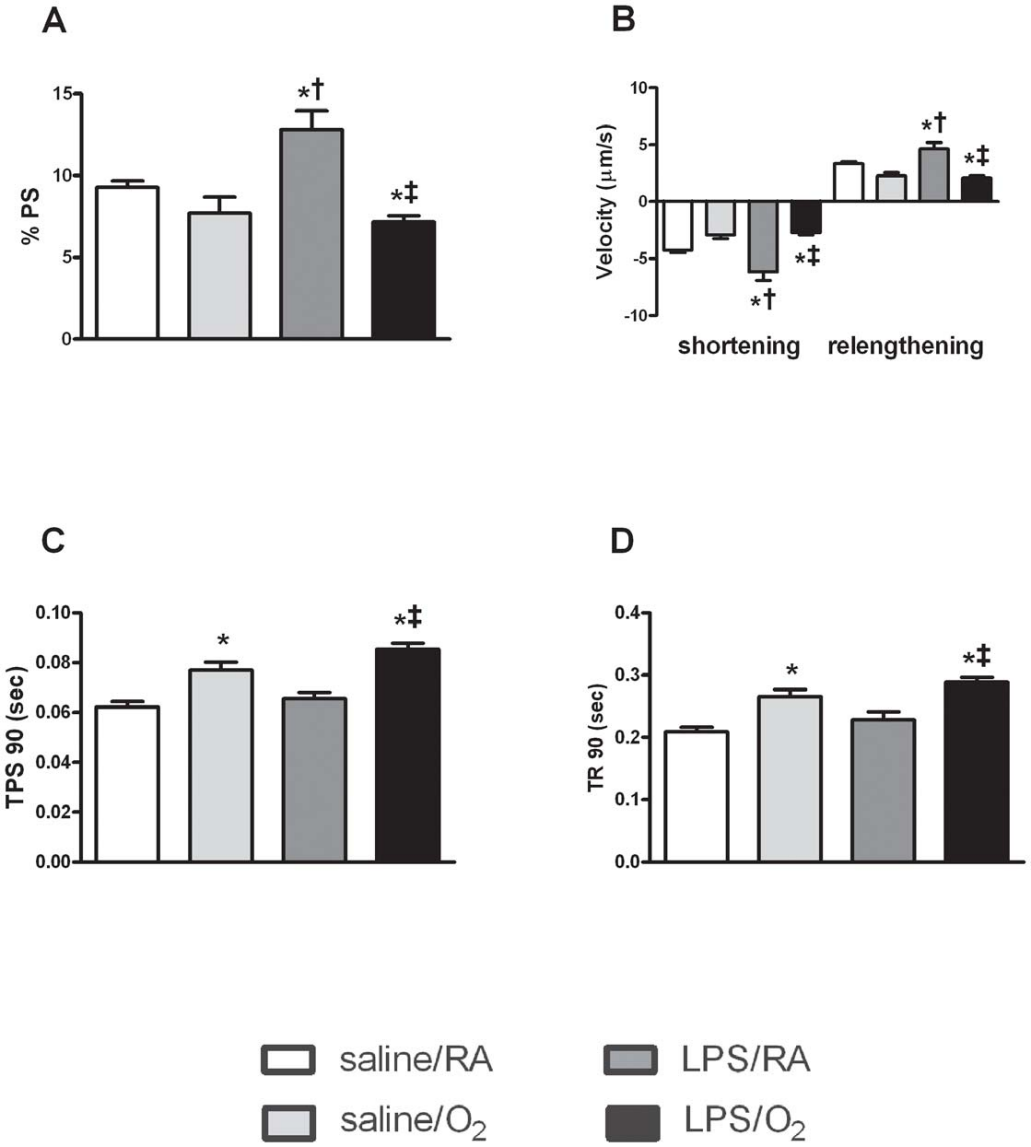
*In vitro* cardiomyocyte function in saline/RA and LPS/O_2_ exposed mice at 8 weeks of age. (A) % Peak shortening (% PS) was increased in the LPS/RA exposed mice. (B) Shortening velocity (Dep) and relengthening velocity (Rel) was significantly increased in cardiomyocytes isolated from LPS/RA exposed mice and decreased in LPS/O_2_-exposed mice compared to saline/RA controls. (C) Time-to-90% shortening (TPS 90) was significantly increased in cardiomyocytes isolated from saline/O_2_ and LPS/O_2_-exposed mice, indicating systolic dysfunction at the cellular level. (D) Time-to-90% relengthening (TR 90) was significantly increased in myocytes from saline/O_2_ and LPS/O_2_-exposed mice, indicating significant diastolic dysfunction at the cellular level. Data were analyzed using one-way ANOVA and Bonferroni post hoc, *p<0.05, n = 20 cells per mouse and three to five mice per group. p<0.05 compared to saline/RA (*), saline/O_2_ (†), or LPS/RA (‡) exposed mice.

Assessment of cardiomyocyte function was performed on isolated cells obtained from mice at 8 weeks of age. Cell shortening (%PS) at 1 Hz was increased ∼40% in cardiomyocytes isolated from the LPS/RA compared to all other groups (Figure 2A) and was associated with increased maximal shortening and relengthening velocities (±dL/dt)(Figure 2B). Interestingly, %PS and velocities (±dL/dt) were not different between the saline/O_2_ and the saline/RA controls, however both the time to 90% contraction and relengthening were longer. In contrast, contractility was decreased by ∼20% in the LPS/O_2_ group and was associated with an increase in time to 90% contraction (TPS 90; Figure 2C) and impaired maximal shortening velocity (Figure 2B). Furthermore, increased time to 90% relengthening (TR 90; Figure 2D) and decreased maximal relengthening velocity (Figure 2B) were also observed.

### α-Myosin Heavy Chain (α-MHC) and β Myosin Heavy Chain (β-MHC) protein measurements

**Figure 3:**
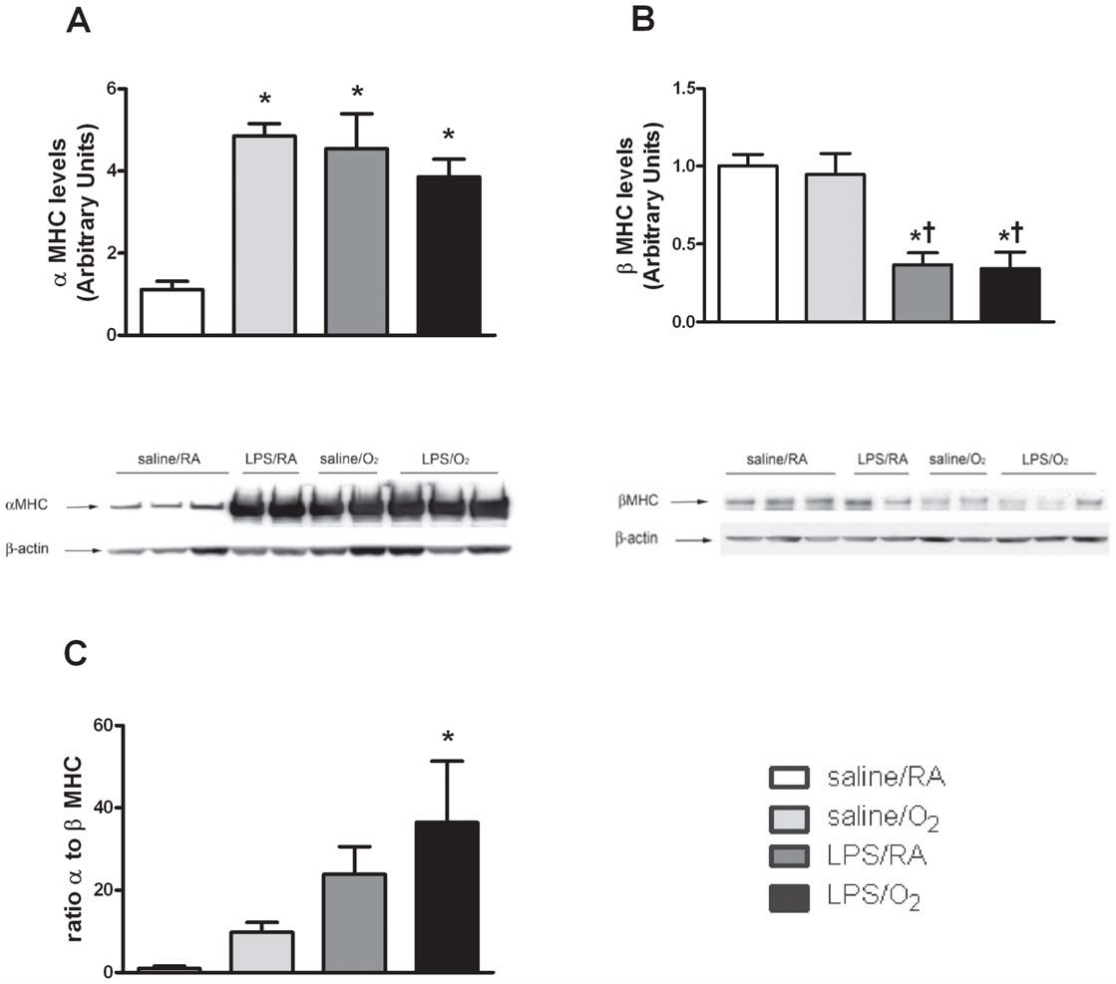
MHC protein contents in LV tissues at 8 weeks of age. Representative Western blots and quantified data indicating changes in α-MHC and β-MHC protein contents due to LPS, O_2_ or combined treatments. Data were analyzed using one-way ANOVA and Bonferroni post hoc, n = 5 mice per group, p<0.05 compared to saline/RA (*) exposed mice.

LV α-MHC protein content was significant increased in all treatment groups compared to saline/RA exposed mice (Figure 3A). Conversely, β-MHC protein contents were not affected in saline/O_2_ but significantly reduced in LPS/RA and LPS/O_2_, exposed mice (Figure 3B). The ratio of α- to β-MHC indicates a pronounced shift toward the more energy consuming α-MHC in the LPS/O_2_ exposed mice (Figure 3C).

### Connexin-43 immunohistochemistry and protein

**Figure 4:**
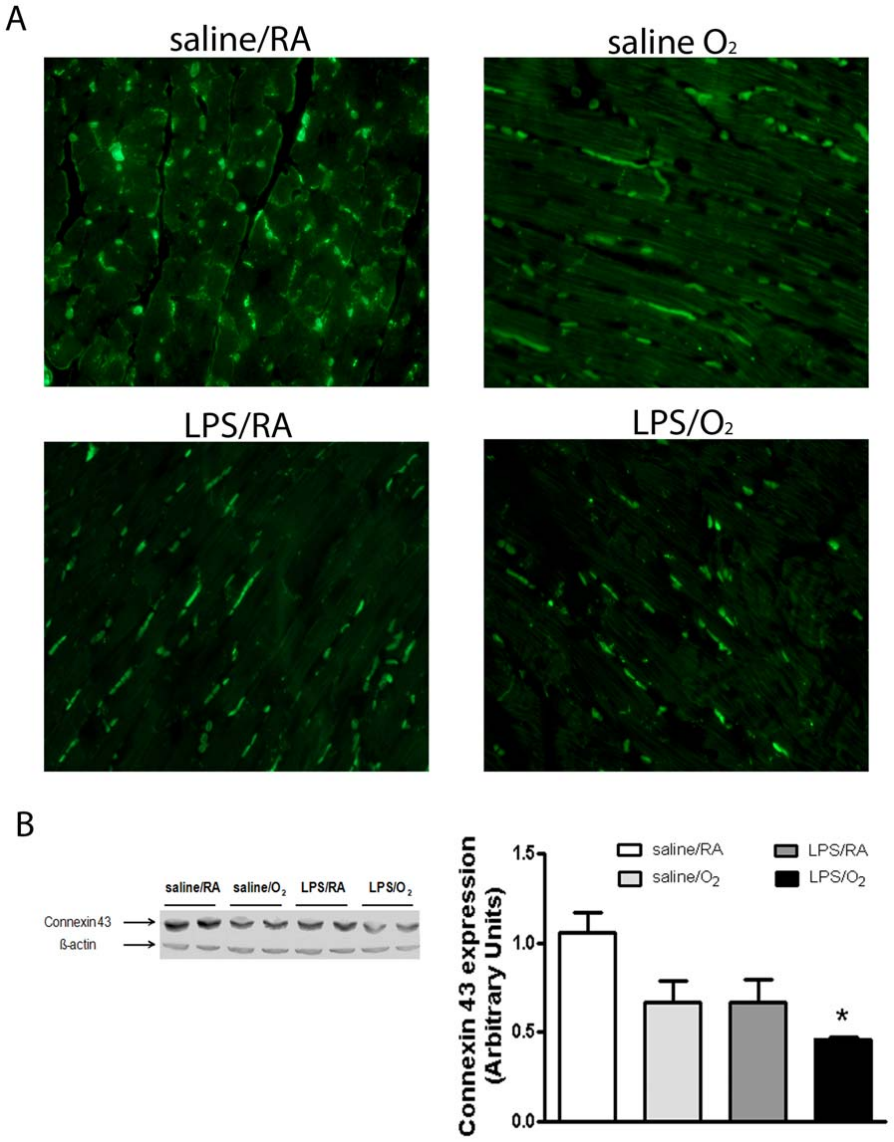
Connexin-43 proteins in LV tissues at 8 weeks of age. Representative confocal images showing reduced numbers of connexin-43 positive gap junctions and CX-43 lateralization in saline/O_2_, LPS/RA, and LPS/O_2_ compared to saline/RA-exposed mice (Figure 6A). Representative Western blots and quantified data indicating significantly reduced connexin-43 content in LPS/O_2_ compared to saline/RA exposed mice (Figure 6B). Data were analyzed using one-way ANOVA and Bonferroni post hoc, n = 5 mice per group, p<0.05 compared to saline/RA (*) exposed mice.

Histological assessments revealed fewer connexin-43 positive gap junctions, with positive staining more lateralized in all treatment groups compared to saline/RA exposed mice (Figure 4A). LV connexin-43 protein content was reduced in all treatments compared to saline/RA exposed mice (Figure 4B).

### Nox4 and VEGFA protein expression

**Figure 5:**
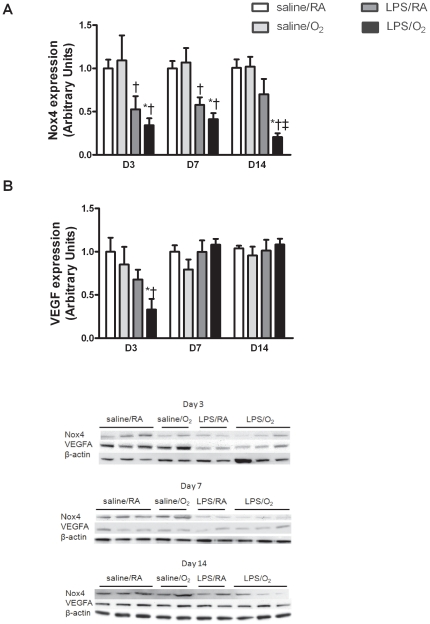
Western blot assessments of Nox4 and VEGFA protein levels. Representative Western blots and quantified data indicating changes in Nox4 and VEGFA protein contents due to LPS treatment. Data were analyzed using one-way ANOVA and Bonferroni post hoc, n = 5 mice per group, p<0.05 compared to saline/RA (*), saline/O_2_ (†), or LPS/RA (‡) exposed mice.

Nox4 protein levels were dramatically decreased in both LPS/RA and LPS/O_2_ exposed mice compared to the saline groups at days 3 and 7 (Figure 5A). At day 14, the LPS/RA mice had compensated and NOX 4 levels were no longer different than the saline groups but different than LPS/O_2_ (Figure 5A), while Nox4 expression in the LPS/O_2_ groups was persistently decreased. Decreases in Nox4 protein levels in the LPS-treated mice coincided with decreases in VEGFA expression at day 3 however, at days 7 and 14, there was no significant difference in VEGFA among the groups (Figure 5B).

### Capillary Density in the LV myocardium

**Figure 6:**
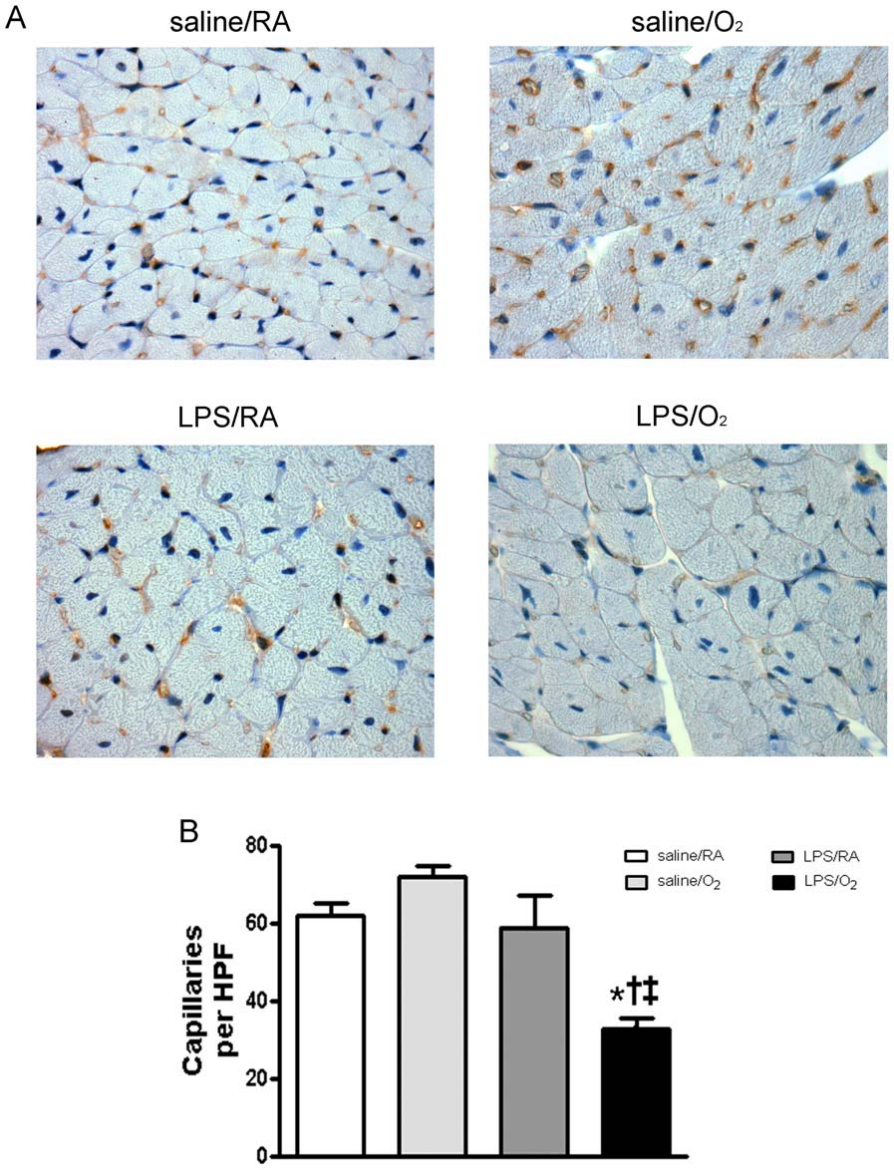
Capillary density was assessed in LV tissues by immunohistochemistry. Images of tissue sections were immuno-stained for CD31 (Figure 6A). Capillary numbers were counted in 5 high power fields (HPF) per slide and n = 3 mice per group (HPF = 22,000 µm^2^). Data were analyzed using one-way ANOVA and Bonferroni post hoc, p<0.05 compared to saline/RA (*), saline/O_2_ (†), or LPS/RA (‡) exposed mice.

Capillary density was assessed in LV tissue sections by immunostaining with CD31 (brown staining indicates vessels <50 microns in diameter, Figure 6A). No apparent abnormalities in endothelial structure within the vessels were observed in histological analyses. Capillary counts revealed fewer capillaries in the LPS/O_2_ exposed mice than in all other exposure groups (Figure 6B).

## Discussion

This study provides the first evidence that the combination of systemic maternal inflammation and neonatal hyperoxia exposure creates synergistic responses in the developing rodent, resulting in prolonged, adverse LV structural and functional changes. Furthermore, these adverse changes are manifested as persistent LV systolic dysfunction *in vivo* and both systolic and diastolic dysfunction *in vitro*.

Maternal inflammation was documented by measurement of TNFα, IL-1β in maternal lung tissues and KC in the plasma of the dam through the remainder of gestation. In the present study, all dams that gave birth had similar litter sizes and pup weights. However, at 8 weeks of age, the LPS/O_2_ exposed mice weighed less than all other groups (Table 1), indicating a developmental impact of early exposures that was not manifested until adulthood. Others have described cardiac dysfunction in response to multiple maternal insults during fetal development with and without a reduction in birth weight.[25], [26] However, our findings are consistent with studies by Rueda-Clausen *et al.* [18] which observed lower body weights at 12 months of age in rats born to hypoxia exposed dams.

Adverse maternal environments can induce LV remodeling leading to cardiac dysfunction.[27], [28], [29], [30], [31], [32] Studies by Bal *et al.* have observed decreased LV weight in response to neonatal dexamethasone treatment which resulted from inhibition of mitosis and a reduced number of cardiomyocytes at adulthood.[33] In the LPS/O_2_ exposed mouse pups, lower LV weights were observed at 8 weeks, with this difference remaining even after correcting for body weight (Table 1). Neither total liver weight nor liver weight normalized to body weight was different in any group indicative of no overall alterations in body weight (Table 1). The decreased LV weight was consistent with a decrease in LV posterior wall thickness indicating lower muscle mass and was most pronounced in the combined treatment group suggesting synergism between the maternal LPS and neonatal hyperoxia exposure.

Lower LV weights correlated with increased LV end systolic diameter and decreased FS in LPS/O_2_ exposed mice (Figure 1) and indicate a systolic dysfunction that is distinct from diastolic dysfunction previously reported.[18], [34], [35] Systolic dysfunction is indicative of more severe cardiac dysfunction, and has not been previously described in animal models of maternal/neonatal exposure. Interestingly, these data are consistent with cardiac dysfunction observed at 5 years of age in humans that were born small for gestational age.[36]

The systolic dysfunction observed was further investigated at the cellular level in isolated cardiac myocytes. Interestingly, LPS alone actually increased cardiac myocyte contractility in response to electrical pacing at 1 Hz (Figure 2) and increased the kinetics of both shortening and relaxation. In adult rodents, the myocardial consequences of LPS-induced sepsis are hypotension and cardiac hypocontractility.[37], [38] However, the fetus is able to respond immediately and adapt to an adverse uterine environment e.g. maternal sepsis, for self-preservation.[39] In the present study, this adaptation persists through adulthood, so called predictive adaptation. Conversely, the LPS/O_2_ myocytes exhibited impaired contractile performance as evidenced by the kinetics of shortening and relaxation (Figures 2C, 2D), which indicates that the fetal adaption was not sufficient to sustain contractility if mice were subsequently exposed to oxygen. Collectively, these data indicate that the combination of LPS and O_2_ exposure causes a unique phenotype which may account for the contractile defects observe *in vivo*. Often the results obtained in cardiomyocyte function are different than what is observed *in vivo*. This was the case in our study and is potentially due to alterations in the functional architecture of the heart. Interestingly, it is apparent that the cardiomyocyte is specifically targeted by LPS treatment as evidenced by the times required for 90% contraction or relaxation which is indicative of diastolic dysfunction. The slower rates of myocyte relaxation *in vitro* were not yet manifested as diastolic dysfunction *in vivo*. This may be attributed to several factors, including the presence of neurohormonal influences *in vivo* that were not present in the isolated cell culture, alteration in intercellular electrical mechanical coupling, and or changes in the composition of the extracellular matrix. However, preliminary studies indicated no evidence of fibrosis or changes in matrix composition.

One mechanism that may underlie the decreased contractile kinetics in LPS/O_2_ cardiomyocytes is the myosin heavy chain isoform switching. In the rodent heart, the α-MHC isoform expression predominates in healthy cardiac tissue, and a transition to greater β-MHC levels occurs during pathological LV remodeling and experimental heart failure in a variety of adult animal models.[40] Specific nutritional deprivations have also been shown to cause alterations in cardiac structural proteins and result in ventricular dysfunction.[41] However, at 8 weeks of age, significant increases in α-MHC, which exhibit a higher ATPase activity and sliding velocity than the β-MHC isoform [42], were evident in either single LPS or O_2_ treatments or in the combined treatment group (Figure 3). Surprisingly, β-MHC was decreased by LPS in LV tissues and unchanged in the other group (Figure 3B) which was contrary to other animal models of cardiac diseases. Furthermore, a shift toward α-MHC in the LPS/O_2_ exposed mice was further evident when relative ratios of α- to β-MHC were analyzed (Figure 3C). Taken together, these alterations in α-MHC/β-MHC levels in our model identify a unique alteration in MHC isoform expression and may partially explain the reduced contractile kinetics in the LPS/O_2_ isolated cardiac myocytes. However the shift toward increased α-MHC, a more energy demanding and greater force generating isoform, is likely a compensatory mechanism by which the mice are able to maintain homeostasis is the face of contractile deficiency. [43], [44]

Decreased levels and/or dislocation of connexin-43 positive gap junctions are strongly correlated with heart failure.[45], [46] We observe a translocation and lateralization of connexin-43 gap junctions in all treatment groups that was most pronounced in LPS/O_2_ compared to the saline/RA exposed mice (Figure 4). The change in localization corresponds to lower overall connexin-43 protein levels in the LPS/O_2_ exposed mice and is likely contributing to the contractile deficits we observe. Lateralization is thought to occur when connexin-43 is hyperphosphorylated and just prior to degradation. This change in location and phosphorylation state prevents connexin-43 proteins from integrating into the gap junction structure and contributes to the slowing of longitudinal conduction velocity.[47]

Histological examination of whole heard sections revealed no difference in the size and shape of the heart but a decreased number of capillaries was noted. Capillary formation in the myocardium is driven largely by the paracrine expression of vascular endothelial growth factor (VEGF). VEGF expression and activity is regulated by changes in oxygen tension and the formation of reactive oxygen species (ROS) which are produced in response to inflammation as well as hyperoxia exposure. In the heart, ROS are readily produced in part by the activity of NADPH oxidases (NOX). In particular, Nox4 facilitates capillary formation by stabilizing hypoxia inducible factor 1 and increasing the expression of VEGF, resulting in angiogenic activity.[48] In the present study, Nox4 expression was severely depressed in the heart of mice exposed to LPS at days 3 and 7 and remained depressed in the LPS/O_2_ exposed mice even at 14 days (Figure 5A). The decreases in Nox4 expression in the LPS/O_2_ exposed mice were accompanied by the early deficits in VEGF expression at day 3 (Figure 5B). Although VEGF expression returns to control levels by day 7, the early developmental deficiency in VEGF expression at a potentially critical time point could permanently effect capillary formation leading to reduced density as evident at 8 weeks of age (Figure 6). Although our findings indicate an acute and temporally defined VEGF deficit, the phenotype observed in LPS/O_2_ exposed mice is remarkably similar to that reported by Giordano et al. [49] in a cardiomyocyte-specific VEGFA knockout. They reported contractile dysfunction, ventricular wall thinning and decreased capillary density without changes in the major vessels.

The phenotype of the LPS/O_2_ exposed mice is complex. Our data indicate that these mice exhibit ventricular wall thinning, decreased cardiac contractility, and impaired myocardial capillary formation. The collective pathologies could result from a single defect early in development, however this is unlikely given that the maternal inflammation is induced at E16, well into the third trimester of development after most of the key events in heart formation, and the hyperoxia exposure occurs after birth. It is interesting, however, that the VEGFA knockout mice reported by Giordano [49] have similar phenotypes resulting from ablation of a single gene. Obviously, expression and regulation of VEGFA in our models warrants further investigations.

In conclusion, the present study highlights the impact of the perinatal environment on cardiac development and its effects on structural and functional changes in the adult heart. The extremely preterm infants born to mothers with systemic inflammation and subsequently treated with oxygen therapy have survived this ordeal only in the last ∼20 years. The phenotype observed in our model is severe and may exaggerate the disease propensity in human infants however, Bassero et al. have observed long QT intervals in ex-extremely preterm infants as early as 20–21 years of age.[50] Our model offers a basis to begin to understand the mechanisms behind the pathologies that this unique population is likely to face. The impact of an adverse perinatal environment on cardiovascular health may be not be evident until additional risk factors are introduced in adulthood and could be the underlying source of idiopathic cardiac events, specifically in this population. The need for better understanding the long-term consequences of the fetal and neonatal environment is obvious and could provide alternative approaches to the development of interventional strategies.

## Methods

### Animal Model

All animals were handled in accordance with NIH guidelines and protocols were approved by the Institutional Animal Care and Use Committee at the Research Institute at Nationwide Children's Hospital. Male and female C3H/HeN mice were paired and the presence of a vaginal plug was designated as embryonic day one (E1). In preliminary studies, we investigated in the highest LPS dose that if injected to pregnant dams on E16 resulted viable litter without differences in size or life born pups. On E16, pregnant dams were injected with LPS (80 µg/kg i.p., serotype 0111:B4 Calbiochem, #437627), or an equal volume of saline. After birth 2 liters of newborn mice born to saline or LPS injected dams were pooled and redistributed randomly to two dams (with similar E16 treatments) and placed in separate cages. One dam and litter was placed in a plexiglass chamber containing a 10 L/min flow of 85% O2 while the corresponding dam and litter were placed in room air (RA). One litter of pups was exposed to 85% O_2_ for 2 weeks (saline/O_2_, LPS/O_2_) and then returned to room air (RA) while the other litter of pups was maintained in RA (saline/RA, LPS/RA). The dams were switched every 24 h to prevent oxygen toxicity. Twenty-four hours of hyperoxia exposure was designated as day 1.

### Echocardiography studies

At 2 and 8 weeks of age, mice were anesthetized with isoflurane and placed in a supine position on a physiological warming pad. Echocardiographic evaluations were performed using a VisualSonics Vevo 2100 Ultra High Resolution *In Vivo* Imaging System (VisualSonics, Toronto, ON, Canada). Scanning was performed at a frequency of 20MHz and three measures at different cardiac cycles were assessed and used for analysis. M-mode images were obtained in the parasternal short axis view at the level of the papillary muscles to assess left ventricular (LV) end systolic diameter (LVES_d_) and LV end diastolic diameter (LVED_d_). Stroke volume (SV) was determined using Doppler flow Velocity-Time Integral (VTI) at the LV outflow tract (LVOT) and the aortic diameter (Ao), (LVOT^2^ * 0.785 * Ao VTI). Cardiac output was calculated from stroke volume multiplied by heart rate (SV*HR). Systolic function was assessed using M-mode calculations of fractional shortening (FS = LVEDd-LVES_d_/LVEDd) LV end diastolic volume (LVEDV) was calculated from LVED_d_ (7/(2.4+LVED_d_)*LVED_d_^3^). LV end systolic volume (LVESV) was calculated from LVES_d_ (7/(2.4+LVES_d_)*LVES_d_^3^) and ejection fraction (EF = (LVEDV-LVESV)/LVEDV*100).

### Isolation and functional assessments of LV cardiomyocytes

At 8 weeks of age, left ventricular cardiomyocytes were isolated by retrograde aortic perfusion with liberase and cultured until the time of experiment as described previously.[51] Cardiomyocytes adherent to laminin coated imaging chambers were loaded onto the stage of an inverted microscope (Olympus IX-70, Olympus Corporation, Tokyo, Japan). Cells were perfused with heated contractile buffer (131 mM NaCl, 4 mM KCl, 10 mM HEPES, 1 mM CaCl_2_, 1 mM MgCl_2_, and 10 mM Glucose) at 37°C, and stimulated with a suprathreshold voltage using two platinum wires at a frequency of 1.0 Hz. Myocyte mechanics (twitches) were assessed using a Myocyte Calcium Imaging/Cell Length System (Ionoptix, Milton, MA). Data were acquired with a Soft Edge MyoCam® system (IonOptix Corporation, Milton, MA, USA). Cell shortening (a measure of cellular systolic function) and re-lengthening (a measure of cellular diastolic function) as well as time for each were measured on individual cells using video recorded images. These data are reported as peak shortening normalized to baseline sarcomere length (%PS), time-to-90% shortening (TPS 90), time-to-90% relengthening (TR 90), and the maximal velocities of sarcomeric shortening and relengthening (±dL/dt).

### Immunoblot analyses of LV tissue

LV protein lysates were separated by SDS-PAGE and transferred to PVDF membranes. Membranes were probed with antibodies to α-MHC (1∶1000, Genway, 20-272-191956, San Diego, CA), β-MHC (1∶500, Santa Cruz, sc71575, Santa Cruz, CA), Nox4 (1∶5000, a generous gift from Dr. Reto Asmis), VEGFA (1∶500, Santa Cruz, sc-152, Santa Cruz, CA), connexin-43 (1∶5000, Santa Cruz, sc-9059, Santa Cruz, CA), followed by their corresponding secondary antibodies. Bands were visualized using ECL detection and quantified with densitometry using ImageQuant software, v5.0 (Molecular Dynamics). Band densities were normalized to β-actin (1∶10000, Abcam, ab6276, Cambridge, MA) or total ERK protein (1∶10000, Abcam, ab16869, Cambridge, MA).

### Immunohistochemistry

Heart sections (5 µm) were deparaffanized, blocked, and treated with CD31 antibody (1∶100, Santa Cruz, sc-1506, Santa Cruz, CA) or connexin-43 (1∶100, Santa Cruz, sc-9059, Santa Cruz, CA). Secondary antibody and ABC reagents including 3,3′-Diaminobenzidine (DAB) were used as the peroxidase substrate for CD31 and hematoxylin was used to counterstain. For connexion-43 staining, Alexa Fluor 488 (Invitrogen, Carlsbad, CA) was used as the secondary antibody and viewed with fluorescent microscopy. Unique photomicrographs from each treatment were recorded at (100×) and capillaries counted by an investigator blinded to group assignment.

### Statistical analyses

Data are presented as mean ± SEM. Statistical analyses were performed using two-way ANOVA followed by Bonferroni post-hoc analyses. P<0.05 was considered statistically significant. Analyses were performed using GraphPad PRISM 5 (La Jolla, CA).
